# Subjective valuation of Iranian women for screening for gene-related diseases: a case of breast cancer

**DOI:** 10.1186/s12889-023-15568-0

**Published:** 2023-04-11

**Authors:** Zahra Meshkani, Najmeh Moradi, Ali Aboutorabi, Abdosaleh Jafari, Roshanak Shams

**Affiliations:** 1 Department of Health Economics, School of Health Management and Information Science, Iran University of Medical Sciences, Tehran, Iran; 2 Population Health Sciences Institute, Newcastle University, Newcastle upon Tyne, United Kingdom; 3 Department of Health Economics, School of Health Management and Information Science, Iran University of Medical Sciences, Tehran, Iran; 4 Health Human Resources Research Center, School of health Management and Information Sciences, Shiraz University of Medical sciences, Shiraz, Iran; 5 Bone and Joint Reconstruction Research center, Department of Orthopedics, School of Medicine, Iran University of Medical Sciences, Tehran, Iran

**Keywords:** Breast cancer, Genetic screening test, BRCA, Willingness to pay (WTP), Iran

## Abstract

**Background:**

About 5–10% of breast cancer cases are attributed to a gene mutation. To perform preventive interventions for women with a gene mutation, genetic screening BRCA tests have recently been implemented in Iran. The present study aimed to determine Iranian women’s subjective valuation for screening BRCA tests for early detection of breast cancer to help policymakers to make decisions about genetic screening tests for breast cancer and to know the applicants.

**Methods:**

An online survey was completed by women older than 30 years old in Tehran, the capital of Iran in 2021. A hypothetical scenario about genetic screening tests for breast cancer was defined. The subjective valuation for the tests was assessed by a willingness to pay (WTP) using the contingent valuation method (CVM) by payment card. Demographics, history of breast cancers, knowledge, and physiological variables were considered as independent variables, and a logistic regression model assessed the relationship between WTP and the variables.

**Results:**

660 women were included. 88% of participants intended to participate in BRCA genetic screening for breast cancer if it were free. The mean WTP for the tests was about $ 20. Based on the logistic regression, income, family history of breast or ovarian cancer, and positive attitude were associated with WTP.

**Conclusions:**

Iranian women were willing to intend for genetic screening BRCA tests and pay for them as well. The result of the present study is of great importance for policy makers when it comes to funding and determining co-payments for BRCA genetic screening tests. To achieve a high participation rate of women in breast cancer screening plans, a positive attitude should be promoted as a psychological factor. Educational and informative programs can help.

**Supplementary Information:**

The online version contains supplementary material available at 10.1186/s12889-023-15568-0.

## Background

Breast cancer is the most common cancer among women in the world [1] and also among Iranian women [2]. In 2020, 11.7% (2,261,419 cases for both sexes and all ages) of all cancers worldwide were due to this disease [3] Studies have shown that about one and a half million women contact the disease every year [4].

The mean age for breast cancer in Iranian women is 45 years [5] and the incidence rate is increasing [6]. Valipour, A.A et al., have predicted that the number of new cases and the mortality rate of the disease in Iran will increase by 2035 [7].

Breast cancer imposed a great economic burden on the health care system. Several studies have been conducted on the costs of breast cancer worldwide. For example, based on a systematic review by Sun et al., the average treatment cost for breast cancer at stages I to III/IV in 2015 were $29,724, $39,322, $57,827, and $ 62,108, respectively [8]. The economic burden of breast cancer in Iran was estimated to be $947,374,468 [9].

Screening can detect the disease early, saving many years of women’s lives and reducing treatment costs for the health system [10].

About 90–95% of breast cancers are sporadic and depend on demographic, breast-related, hormonal, reproductive, and lifestyle [11]. The rest is attributed to heredity and gene mutations. A woman with the origin of the gene mutation is classified as a high-risk woman [12]. Although there are several genes affected by hereditary breast cancer, the BRCA genes have been recognized as the most important [13].

Women can have a mammogram as a screening method from the age of 40. This method has reduced breast cancer rates by 20% [14]. It is recommended for low-risk women, while genetic screening tests have been defined for high-risk women [12]. Identifying the inherited cancer risk can help detect cancers early and preventive interventions in high-risk women [15].

In Iran, screening methods such as mammograms and genetic screening tests are performed as a new technology [16]. More than half of Iran’s population is women. The screening of breast cancer can save money and improve the quality of life of patients. A successful screening program depends on identifying resources and the proportion of women as care recipients or beneficiaries and their valuation of the program.

To our knowledge, this is the first study in Iran that aims to determine the subjective valuation for screening BRCA tests, as it is a new technology for early detection of breast cancer.

## Methods

### Study design and participants

This was an online cross-sectional population-based survey. The study was conducted from July, 4 to August 30, 2021. The study population was Iranian women older than 30 years in Tehran who had internet access. We focused on Tehran where occur the most cases of breast cancer [17]. A convenience sampling method was performed. Convenience sampling is a type of sampling in which the researcher uses a sample that does not provide for every member of a target population to participate in a study, but rather for participants to be selected by the researcher or to choose to participate themselves. Convenience sampling is used for population-based studies as it is incredibly fast, uncomplicated and inexpensive. In many cases, members are easily approachable to attend the rehearsal [18]. During the period of our study, the Covid 19 pandemic was triggered and the policy of social distancing and quarantine was enforced. Given that many Iranians had internet access and our study was also population-based, an online questionnaire was used.

There was no sampling frame and the responses to the questionnaire were completely random.

The questionnaire link was sent to anyone who could only respond anonymously and/or forward it to someone else, so the questionnaire link was sent out randomly. The sample size was defined based on Mitchel and Carson [19].

Women’s subjective valuation for the BRCA genetic screening tests for breast cancer was determined using the WTP. Based on the subject theory of value, value defines an individual’s desire or need for a service or object [20].

### Questionnaire development

The questionnaire was designed based on the studies by Manchanda, R., et al. [21], Thompson, H.S., et al., [22], Helmes, A.W., D.J. Bowen, and J. Bengel [23], and Miron-Shatz, T., et al. [24]. To perform a content validity, the variables in the studies were discussed with the expert panel and a multidisciplinary team of health economists, oncologists, and geneticists.

The designed questionnaire included approximately 50 questions and consisted of six sections: demographic information, personal and family history of breast or ovarian cancer, knowledge of genetic screening tests, perceived risk and worry about developing breast cancer, perceived the risk of genes mutations, and attitude towards performing genetic screening tests.

**Fig. 1:**
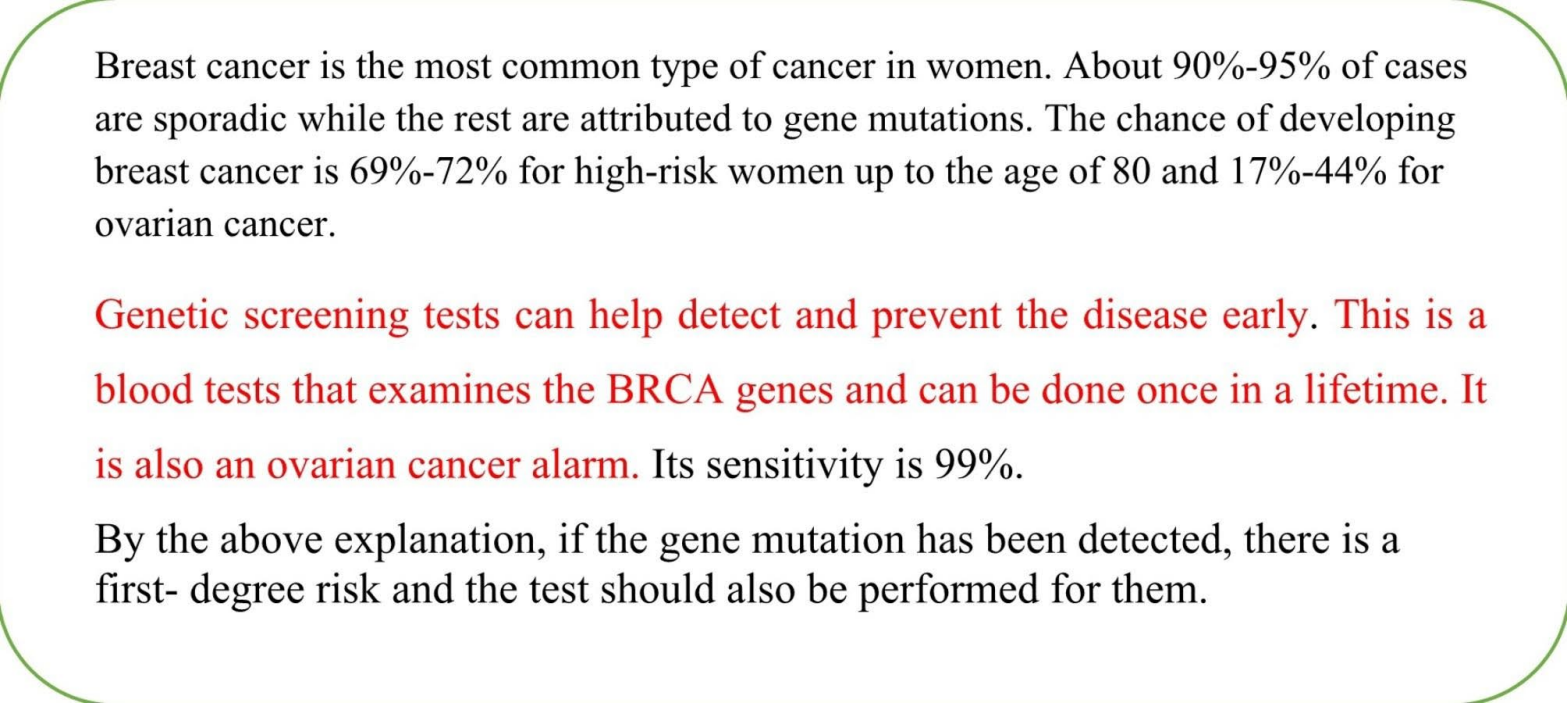
General information about genetic screening testing for breast cancer

**Fig. 2:**
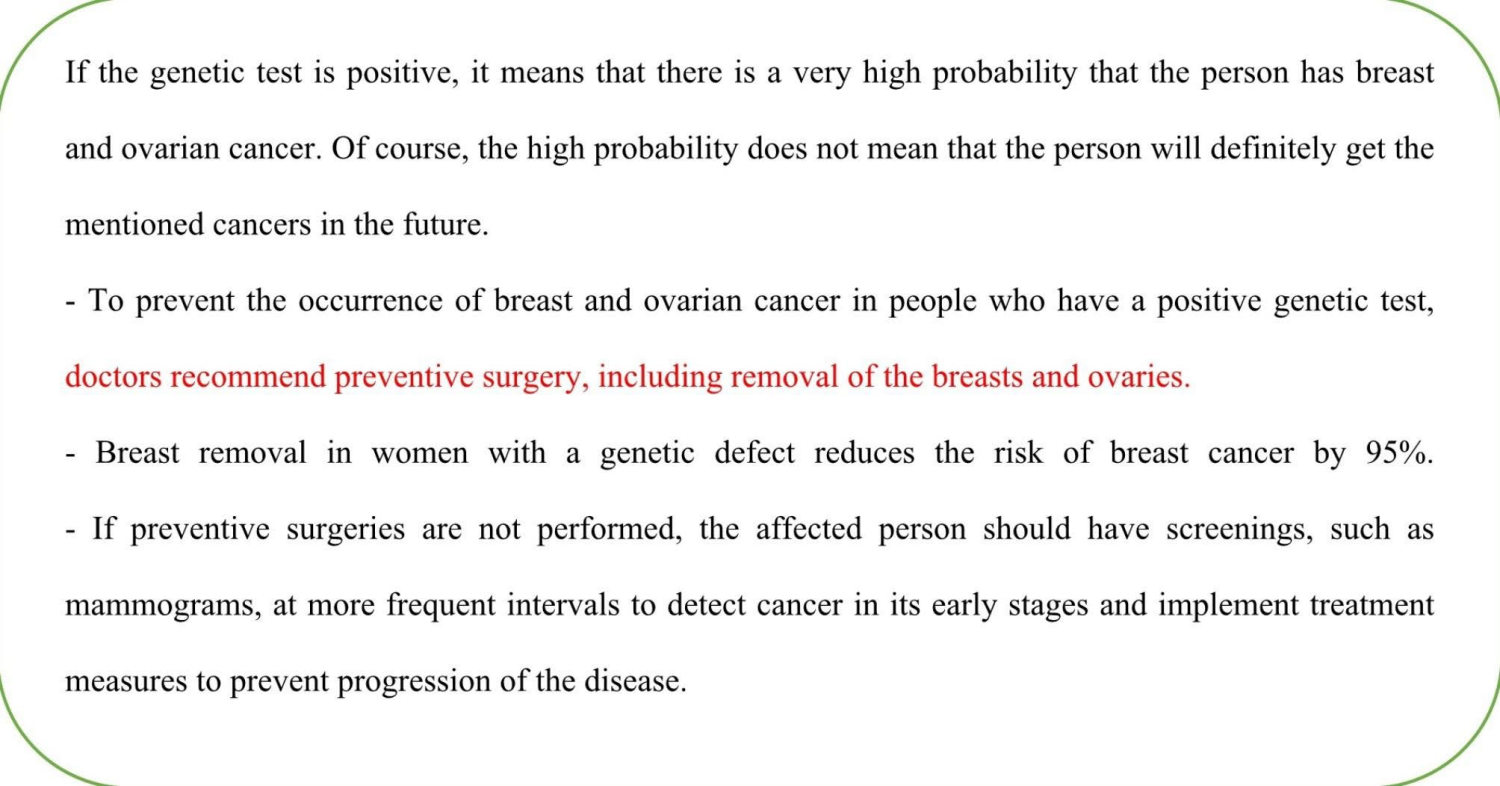
Specific information about genetic screening testing for breast cancer

The independent variables were included as follows:

- **Demographic information**: Age, marital status, education level, occupational status, monthly income, insurance coverage, and the presence of children were considered as demographic parts of the questionnaire, which were asked through closed-ended responses.
- **Personal and family history of breast or ovarian cancer**: This was a “yes” and “no” question where respondents indicated their history of breast or ovarian cancer. The family history question asked about breast or ovarian cancer in the family of blood relatives.
- **Knowledge of genetic risk for breast cancer**: The number of 10 questions about the genetics of breast cancer was considered based on Breast Cancer Genetic Counselling Knowledge Questionnaire (BGKQ) [25], and Kinney, A.Y., et al. [26]. Respondents could choose “True” and “False”. True and false answers were scored 0 and 1, respectively. Women who scored more than half were considered knowledgeable about a gene mutation for breast cancer.
- **Physiological variables**: Perceived risk of developing breast cancer, perceived risk of gene mutation, worry about developing breast cancer in the future, and attitudes towards genetic screening tests were included in this category. They were computed as follows:
  **Perceived risk of developing breast cancer**: The probability of developing breast cancer was asked of respondents who had no history of breast cancer. It was a question by Likert scale question: “I will not get infected at all”, “I will not get infected”, “I am 50% more likely to get it”, “I am more than 50% more likely to get it”, and “I will definitely get breast cancer”.
  **Perceived risk of a gene mutation**: The risk of a gene mutation was asked of respondents who had not tested in the past. It was a Likert scale question with the answers “No gene mutation at all”, “Less than 50% chance of having a gene mutation”, “A 50% chance of having a gene mutation”, and “More than 50% chance of having a gene mutation”.
  **Worry about getting breast cancer in the future**: This question was asked of respondents who had no personal history of breast cancer. It was a Likert scale question: “Not at all worried”, “Not worried”, “Slightly worried”, “Worried”, and “Very worried”.
- **Attitudes towards genetic screening tests**: Based on Manchanda, R., et al. [21], and Kinney, A.Y., et al. [26]. The number of nine sentences about genetic screening tests in women was considered and respondents could choose between “agree”, “disagree”, and “do not know”. Positive and negative responses were scored as 1 and − 1, respectively. For the “do not know” answers, the value of 0 were taken into account. Women who answered more than half of score had a positive attitude towards conducting genetic screening tests for women with breast cancer.
- **Wilingness To Pay (WTP)**
  Subjective valuation of genetic screening tests were determined by a WTP using the CVM. Using a survey design, a hypothetical scenario consisting of general and specific information about genetic testing for women over 30 years of age was presented, and participants were asked their maximum WTP for genetic screening tests as an out-of-pocket (Fig. 1 and Fig. 2).
  Participants indicated their WTP on a payment card scale that ranged from $4 to more than $434 (Table 1). In addition, respondents could indicate their WTP if it was not on the scale. A pilot study was conducted with 60 women over the age of 30 to determine these values using an open-ended WTP question.
  The question was, “If the test is not free, what is the maximum amount you are willing to pay as out of pocket for a genetic test for breast cancer present year?“
  We have converted the indicated WTP in the local currency to USD. Due to wide exchange rate fluctuations, we used a simple moving average of the last 200 days to convert values to U.S. dollars. Accordingly, US $1 is equivalent to IRR^1^ 230,000 in the period of the study.

**Table 1:** The payment card for genetic screening tests of breast cancer

$4
$9
$13
$17
$22
$43
$87
$130
$174
$217
$435 and more

### Data collection

Due to the Covid-19 pandemic, an online questionnaire was designed. Social media such as WhatsApp, Telegram, Instagram, and email invitations were used to distribute the questionnaire. At the beginning of the questionnaire, the research objective of the study was explained and respondents were optional to answer the questionnaire. Technical knowledge about genetics of breast cancer was not required and participants were assured that identifying information would remain confidential.

### Data analysis

Descriptive analysis was undertaken for the variables to present the characteristics of the sample size. Minimum, maximum, and mean for WTP were calculated. The proportion of participants who had WTP was estimated for different subgroups: low-risk women (i.e. women who had no family history of breast or ovarian cancer) and high-risk women (i.e. women who had a family history of breast or ovarian cancer), different income levels, and attitudes towards genetic screening tests.

WTP was classified into 0 and 1 for participants who did not have WTP and for participants who did have WTP, respectively. The relationship between WTP and the independent variables was assessed using a logistic regression model. All variables were included in the original logistic regression model. Variables whose p < 0.05 were included in the final logistic model. An alpha level of 0.05 was considered significant in the final model. Odds Ratio, standard error, confidence interval, log likelihood, and chi2 test were reported. Data analysis was conducted using STATA 16 software.

## Results

3844 persons viewed the online questionnaire. The response rate for the questionnaire was 48%. The average response time of the questionnaire was 11:54 min. Then the number of 1100 persons completed the questioner. Men as well as women younger than 30 years were excluded the study. After data cleaning, finally 660 women older than 30 years old were included the study.

### Respondent characteristics

The characteristics of respondents are presented in Table 2. Respondents were on average 40 (SD = 7) years-old. Only 16.8% of the participants were single. About 85% of the women had children. About 11.7% of the participants had postgraduate education. More than half of the respondents (about 57%) were employed. About 40.5% of the women ran a household and did not report a monthly income. About 9% of the respondents had no insurance coverage.

Less than 2% of respondents had a history of breast and ovarian cancer. Less than 1% of respondents had a history of other cancers. About 29% and 9% of the respondents had a history of breast and ovarian cancer in their first and second-degree relatives, respectively. For other kinds of cancers, it was about 48%.

About 19% of the respondents were not worried about developing breast cancer. About 21.7% of the respondents did not consider themselves at risk of breast cancer, although about 51% of them had indicated a probability of having a gene mutation for breast cancer.

Genetic screening BRCA tests are unknown among the participants and they had low knowledge of genetic risk for breast cancer. Although about 25% of the respondents had high knowledge, more than 80% of them had a positive attitude towards performing the tests.

**Table 2:** The respondent characteristics

Variable		Frequency (%)
Participants		660 (100)
Age	30–40 years-old	392 (59.4)
	41–50 years-old	207 (31.4)
	Older than 50 years-old	61 (9.2)
Marital status	Single	111 (16.8)
	Married	549 (83.2)
Education	Elementary, middle school, high school	45 (6.8)
	Diploma	102 (15.5)
	Associate and Bachelor	269 (40.8)
	MSc and Ph.D.	244 (37)
Occupation status	Professional	375 (56.8)
	Retired	21 (3.2)
	Not working, unemployed	264 (40)
Self-reported monthly income	No income	267 (40.5)
	Less than US$ 86	24 (3.6)
	Between US$86–160	34 (5.2)
	Between US$160–217	53 (8)
	Between US$217–260	71 (10.8)
	Between US$260–347	93 (14.1)
	More than US$347	118 (17.9)
Insurance coverage	No	62 (9.5)
	Yes	598 (90.5)
Children	Yes	466 (70.6)
	No	194 (29.4)
Had mammography or sonography	Yes	440 (66.7)
	No	220 (33.3)
Self-history of breast or ovarian cancer	Yes	11 (1.7)
	No	649 (98.3)
Self-history of other kind of cancers	Yes	5 (0.8)
	No	644 (97.6)
Family history in breast cancer	Yes	194 (29.4)
	No	436 (66.1)
	I do not know	30 (4.5)
Family history in ovarian cancer	Yes	54 (8.2)
	No	533 (80.8)
	I do not know	69 (10.5)
Family history in other kind of cancers	Yes	225 (47.8)
	No	284 (43)
	I do not know	61 (9.2)
Perceive risk of developing breast cancer	I do not get infected at all	143 (21.7)
	I will not get infected	239 (36.9)
	I will be 50% more likely to get it	253 (38.3)
	I will be more than 50% more likely to get it	16 (2.4)
	I will definitely get breast cancer	2 (0.3)
Worry about getting breast cancer in the future	Not worried at all	127 (19.2)
	Not worry	183 (27.7)
	Slightly worried	50 (7.6)
	Worried	265 (40.2)
	Very worried	28 (4.2)
Perceive risk of gene mutation	Not have gene mutation at all	304 (47.35)
	Less than 50% chance of having a gene mutation	247 (38.47)
	A 50% chance of having a gene mutation	75 (11.68)
	More than 50% chance of having a gene mutation	16 (2.49)
Knowledge of genetic tests	Less than 5	372 (56.4)
	= 5	121 (18.3)
	More than 5	167 (25.3)
Attitude about genetic screening tests	Positive attitude	596 (90.3)
	Negative attitude	64 (9.7)
Intention of genetic screening tests	Yes	581 (88)
	No	79 (12)

### WTP and associated factors

**Fig. 3:**
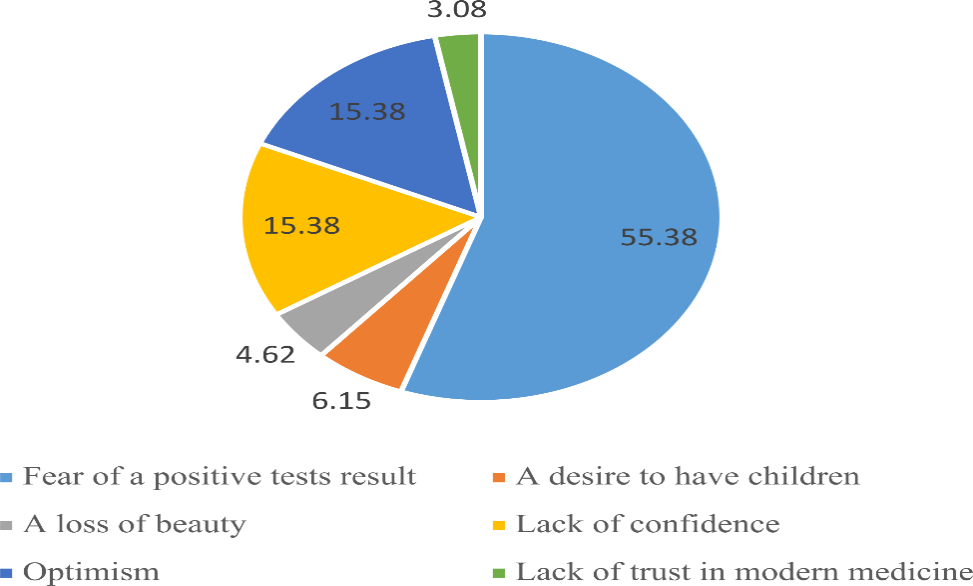
The reasons for withdrawing the genetic screening tests for breast cancer

More than 88% of the respondents had an intention of the genetic screening tests. Fear of a positive test result was the reason for withdrawing genetic screening tests for breast cancer in about half of the women who were not willing to pay for the test, and optimism was the second most common reason. The reasons for withdrawing the tests are shown in Fig. 3.

The mean WTP for the tests was $ 20 (equal to 4,600,000 IRR). The minimum and maximum were $ 0.43 (equal to 98,900 IRR) and $ 434 (equal to 99,820,000 IRR), respectively. The amount of WTP for the tests was less than $ 4 (equal to 920,000 IRR) and between $ 4- $ 22 (equal to 920,000 IRR- 5,060,000 IRR) for 40.65% of the respondents.

**Fig. 4:**
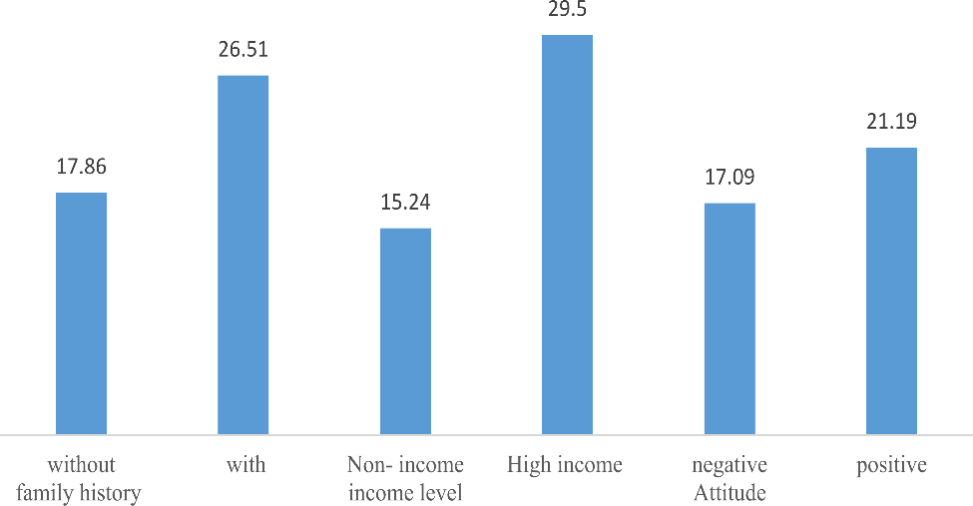
The mean WTP (US$) in different subgroups

The WTP was calculated in different subgroups (Fig. 4). The WTP for the tests was higher in high-risk women (i.e. women with a family history of breast or ovarian cancer) than low-risk women (i.e. women who had no family history of breast or ovarian cancer). The mean WTP of women with a family history was 1.4 times higher than the WTP of women without a family history of these cancers.

WTP for testing was higher among high-income women and women with positive attitudes towards genetic screening tests for breast cancer than among housewives and women with negative attitudes, respectively.

The result of the logistic regression is shown in Table 3. Based on the results, income (adjusted odds ratio (aOR) 0.64, 95% Confidence Interval (CI) 0.47–0.76, P = 0.004), family history of breast or ovarian cancer (aOR 1.84, 95% CI 1.10–3.09, P = 0.020), and attitude towards genetic screening tests (aOR 3.83, 95% CI 2.06–7.1, P = 000) are associated with WTP for genetic screening tests. The overall significance of the regression model was confirmed (P < 0.05). The result of the cross tabulation that shows there is a statistically significant relationship between the attitude, family history of breast or ovarian cancer, and WTP is presented in **Appendix1**.

**Table 3:** The results of logestic reggression by Odds Ratio output

	N (%)	OR	a OR [95% CI]	P-value
Family history in breast and ovarian cancer	619 (96%)	1.23	1.84 (1.10-3.095)	0.020
Attitude about genetic screening tests	637 (99%)	0.559	3.83 (2.065–7.115)	0.000
Monthly income	637 (99%)	0.225	0.64 (0.471–0.869)	0.004
Constant		1.12	3.48 (1.607–7.566)	0.002

Dependent variable: WTP, Log Likelihood= -251.95, Number of observations = 640, LR chi2 = 26.64, Probe > chi2 = 0.00, Pseudo R2 = 0.050

## Discussion

In the present study, the subjective valuation of Iranian women was estimated for genetic screening BRCA tests for prevention or early detection of breast cancer by WTP approach. The results of this study showed that, more than 80% of the participants had the intention to take up the tests. The mean WTP was $ 20 and it was $ 26 for high-risk women. The logistic regression results show that income, family history of breast or ovarian cancer, and attitudes towards genetic screening tests were the factors associated with WTP for the tests.

There have been some studies on WTP for curative as well as screening interventions for breast cancer. Based on the study by Deepa Lalla et al., the mean WTP for patients with metastatic breast cancer to avoid treatment side effects was estimated to be $3894 using a conjoint analysis [27]. Compared with our result for screening for breast cancer, the WTP for treatment interventions was higher than for screening interventions. Our study was subjective and participants had a hypothetical scenario. Consequently, the women’s perceived risk of the disease varied and influenced the participant’s response.

According to Blouin-Bougie, J et al., in 2018, about 90% of women over 35 years in Quebec would like to take genetic screening testing for breast cancer, although the WTP was reported by 57% of them to be less than $100. The contingent valuation method was used for the study [28]. The Iranian women’s WTP for testing was lower than that of women in Quebec. One of the reasons for Iranian women’s low WTP was their expectation of the government to provide subsidies for health services and insurance. Optimism and non-perception of breast cancer risk were reported as other factors leading to low WTP for testing. Age, family history of breast cancer, and income were the factors associated with the genetic tests among women in Quebec. In Iran, although age was not a factor associated with WTP for genetic testing, income and family history were the associated factors.

Based on the results of the study by Wong XY et al., income and education were associated with WTP for genetic screening tests for breast cancer in women aged 40–69 years with no history of breast cancer in a discrete choice experiment in 2018 [29]. In our study, income was associated with WTP, whereas higher educational level and health-related education were not. A higher educational level does not imply expertise in all areas. The women with higher levels of education in our study did not have sufficient information and knowledge in genetics, especially genetic screening tests for breast cancer. Consequently, they had no perceived risk of genetic mutation.

Based on logistic regression, the study by Armstrong et al. in 2000 found that the presence of a family history of breast and ovarian cancer, Jewish ancestry, perceived risk of breast and ovarian cancer, and fear of detection of preventive and curative intervention by the insurance system was associated with the decision to test for BRCA1/2. Interestingly, after counseling, approximately half of the participants opted for genetic screening testing [30]. High-risk women with breast and ovarian cancer had higher WTP for genetic screening tests in our study. Therefore, the results are consistent in this area. The perceived risk of developing breast cancer is higher in high-risk women. Consequently, their WTP would differ from that of low-risk women.

Based on Miron-Shatz et al., a family history of breast and ovarian cancer, Jewish ancestry, and perceived risk of a gene mutation were associated with willingness to genetic screening tests for US women [24] whereas worry as well as the perceived risk of a gene mutation were factors associated with WTP for the test. The WTP of 69% of participants was less than $100 [31]. A family history of breast and ovarian cancer is an important factor in willingness and WTP for genetic screening tests for breast cancer, which the results of our study confirm. The WTP of US women was higher than that of Iranian women. This may be attributed to the difference in the frequency of genetic mutations in the US, especially among those of Jewish descent, as well as higher economic wealth. The US is a country with a private healthcare system, so people’s expectations are more likely to be paid out of pocket. Consequently, their WTP would be higher than that of people who fund their healthcare system from public and government budgets. In the present study, age was not a factor associated with WTP for genetic testing. More than 90% of the participants were younger than 50 years and this may influence the response. Age is a factor in the occurrence of breast cancer but is not related to the gene mutation. Women with gene mutations are at risk for breast cancer up to age 80. If a woman is aware of the risk of developing breast cancer at any age; she will take preventive or surgical interventions to prevent the disease.

It was found out in the current study that attitude is related to WTP for genetic screening tests. Psychological factors such as fear of a positive test result, inclination to have children, fear of losing physical beauty, lack of confidence, and optimism were cited by participants as reasons for unwillingness to take up the tests. This indicates that when these reasons are explained to women by genetic counselors and professionals, their negative attitudes toward genetic testing are reduced and their intention and WTP for testing are also increased. Godard B et al. assessed the factors associated with women’s decision to withdraw genetic screening tests for breast and ovarian cancer in 2007. According to their results, fear of testing positive, travel problems, lack of time, age, personal and health problems, lack of benefits of genetic testing, and lack of insurance coverage were factors associated with withdrawing from the tests [32]. Psychological factors will be very important in the implementation of genetic screening tests for breast cancer, and policymakers can pay attention to this issue to encourage women to undergo the tests.

### Limitations and strengths of the study

The advantage of our study was in assessing the association between knowledge and attitudes toward genetic screening tests and WTP for these tests. Although this was the first study on genetic screening tests for breast cancer in Iran, and the important factors such as physiological variables were identified by the same studies and expert panels, there was some limitations. Less than 2% of the participants had a personal history of breast cancer, so the results might be different if we could focus on women with a personal history of breast cancer. Due to optimism, some women did not perceive the risk of gene mutation, which may affect the results. The majority of Iranian are Muslim, so we could not evaluate the role of religion and race on WTP. As the current study was conducted in Tehran, the results cannot be generalized. Consideration of all Iranian women in future studies may lead to generalization.

## Conclusions

Although less than 10% of breast cancer cases were attributed to a gene mutation, Iranian women were willing to intend and also pay for BRCA genetic testing at an average cost of $ 20. The finding of the present study has major implications for policymakers when it comes to funding and setting copayments for BRCA genetic screening tests. To achieve high participation rates of women in breast cancer screening plans, positive attitudes should be promoted as a psychological factor. Education and information programs can help.

Costing and economic evaluation studies are needed to make a logical decision about which strategy to implement for screening breast cancer attributable to a gene mutation.

## Electronic supplementary material

Below is the link to the electronic supplementary material.

Supplementary Material 1

## Data Availability

All data generated or analyzed during this study are included in this published article.

## Footnotes

1 IRR is the official currency of Iran.

## List of Abbreviations

WTP: Willingness to pay
CVM: Contingent Valuation Method
