# Supplementary material for: Subjective valuation of Iranian women for screening for gene-related diseases: a case of breast cancer
Source: BMC Public Health. 2023 Apr 11;23:667. doi: 10.1186/s12889-023-15568-0 (PMC10088156; doi:10.1186/s12889-023-15568-0)
Supplement: Supplementary file 1 — Supplementary Material 1 [file 12889_2023_15568_MOESM1_ESM.docx]

**Appendix 1**

| **WTP * attitude Cross tabulation** | | | | | |
| --- | --- | --- | --- | --- | --- |
|  | | | attitude | | Total |
|  |  |  | negative | positive |  |
| wta1 | no | Count | 24 | 52 | 76 |
|  |  | Expected Count | 12.2 | 63.8 | 76.0 |
|  |  | % within WTP | 31.6% | 68.4% | 100.0% |
|  |  | % within attitude | 22.6% | 9.4% | 11.5% |
|  |  | % of Total | 3.6% | 7.9% | 11.5% |
|  | yes | Count | 82 | 502 | 584 |
|  |  | Expected Count | 93.8 | 490.2 | 584.0 |
|  |  | % within WTP | 14.0% | 86.0% | 100.0% |
|  |  | % within attitude | 77.4% | 90.6% | 88.5% |
|  |  | % of Total | 12.4% | 76.1% | 88.5% |
| Total | | Count | 106 | 554 | 660 |
|  |  | Expected Count | 106.0 | 554.0 | 660.0 |
|  |  | % within WTP | 16.1% | 83.9% | 100.0% |
|  |  | % within attitude | 100.0% | 100.0% | 100.0% |
|  |  | % of Total | 16.1% | 83.9% | 100.0% |

| **Chi-Square Tests** | | | | | |
| --- | --- | --- | --- | --- | --- |
|  | Value | df | Asymp. Sig. (2-sided) | Exact Sig. (2-sided) | Exact Sig. (1-sided) |
| Pearson Chi-Square | 15.343^a^ | 1 | .000 |  |  |
| Continuity Correction^b^ | 14.070 | 1 | .000 |  |  |
| Likelihood Ratio | 13.026 | 1 | .000 |  |  |
| Fisher's Exact Test |  |  |  | .000 | .000 |
| Linear-by-Linear Association | 15.320 | 1 | .000 |  |  |
| N of Valid Cases | 660 |  |  |  |  |
| a. 0 cells (0.0%) have expected count less than 5. The minimum expected count is 12.21. | | | | | |
| b. Computed only for a 2x2 table | | | | | |

| **WTP * FHBOC Cross tabulation** | | | | | |
| --- | --- | --- | --- | --- | --- |
|  | | | FHBOC | | Total |
|  |  |  | no | yes |  |
| wta1 | no | Count | 54 | 18 | 72 |
|  |  | Expected Count | 47.5 | 24.5 | 72.0 |
|  |  | % within WTP | 75.0% | 25.0% | 100.0% |
|  |  | % within FHBOC | 12.8% | 8.3% | 11.3% |
|  |  | % of Total | 8.4% | 2.8% | 11.3% |
|  | yes | Count | 368 | 200 | 568 |
|  |  | Expected Count | 374.5 | 193.5 | 568.0 |
|  |  | % within WTP | 64.8% | 35.2% | 100.0% |
|  |  | % within FHBOC | 87.2% | 91.7% | 88.8% |
|  |  | % of Total | 57.5% | 31.3% | 88.8% |
| Total | | Count | 422 | 218 | 640 |
|  |  | Expected Count | 422.0 | 218.0 | 640.0 |
|  |  | % within WTP | 65.9% | 34.1% | 100.0% |
|  |  | % within FHBOC | 100.0% | 100.0% | 100.0% |
|  |  | % of Total | 65.9% | 34.1% | 100.0% |

| **Chi-Square Tests** | | | | | |
| --- | --- | --- | --- | --- | --- |
|  | Value | df | Asymp. Sig. (2-sided) | Exact Sig. (2-sided) | Exact Sig. (1-sided) |
| Pearson Chi-Square | 2.967^a^ | 1 | .085 |  |  |
| Continuity Correction^b^ | 2.529 | 1 | .112 |  |  |
| Likelihood Ratio | 3.105 | 1 | .078 |  |  |
| Fisher's Exact Test |  |  |  | .088 | .054 |
| Linear-by-Linear Association | 2.962 | 1 | .085 |  |  |
| N of Valid Cases | 640 |  |  |  |  |
| a. 0 cells (0.0%) have expected count less than 5. The minimum expected count is 24.53. | | | | | |
| b. Computed only for a 2x2 table | | | | | |
